# Differentiation of the Lateral Compartment of the Cochlea Requires a Temporally Restricted FGF20 Signal

**DOI:** 10.1371/journal.pbio.1001231

**Published:** 2012-01-03

**Authors:** Sung-Ho Huh, Jennifer Jones, Mark E. Warchol, David M. Ornitz

**Affiliations:** 1 Department of Developmental Biology, Washington University School of Medicine, St. Louis, Missouri, United States of America; 2 Department of Otolaryngology, Washington University School of Medicine, St. Louis, Missouri, United States of America; Baylor College of Medicine, United States of America

## Abstract

A large proportion of age-related hearing loss is caused by loss or damage to outer hair cells in the organ of Corti. The organ of Corti is the mechanosensory transducing apparatus in the inner ear and is composed of inner hair cells, outer hair cells, and highly specialized supporting cells. The mechanisms that regulate differentiation of inner and outer hair cells are not known. Here we report that fibroblast growth factor 20 (FGF20) is required for differentiation of cells in the lateral cochlear compartment (outer hair and supporting cells) within the organ of Corti during a specific developmental time. In the absence of FGF20, mice are deaf and lateral compartment cells remain undifferentiated, postmitotic, and unresponsive to Notch-dependent lateral inhibition. These studies identify developmentally distinct medial (inner hair and supporting cells) and lateral compartments in the developing organ of Corti. The viability and hearing loss in *Fgf20* knockout mice suggest that *FGF20* may also be a deafness-associated gene in humans.

## Abstract 2

FGF20 signaling in mice is required specifically for the differentiation of cochlear outer hair cells, the cells most often damaged during age-related hearing loss.

## Author Summary

A large proportion of age-related hearing loss is caused by loss or damage to outer hair cells in the organ of Corti. The organ of Corti is a highly specialized structure in the inner ear that is composed of inner hair cells, outer hair cells, and associated supporting cells. Although we understand some of the mechanisms that regulate hair cell versus supporting cell differentiation, the mechanisms that regulate differentiation of inner versus outer hair cells are not known. One potential candidate is fibroblast growth factor (FGF) signaling, which is known to regulate the morphogenesis of many sensory organs, including the organ of Corti. In this study, we find that FGF20 signaling is required at a specific time during development to initiate differentiation of cells in the mouse lateral cochlear compartment (which contains outer hair cells and supporting cells, but not inner hair cells). In the absence of FGF20, mice are deaf, and lateral compartment cells remain undifferentiated and unresponsive to mechanisms that regulate the final stages of differentiation. These findings are significant given the importance of outer hair cells during age-related hearing loss. Our studies also suggest that genetic mutations in FGF20 may result in deafness in humans and that FGF20 may be an important factor for the repair or regeneration of sensory cells in the inner ear.

## Introduction

Congenital hearing loss is one of the most common hereditary diseases, affecting 2–3 infants per 1,000 live births [1]. Acquired age-related hearing loss affects one-third of people over the age of 65 [2]. A large proportion of age-related hearing loss is sensorineural and is caused by loss or damage to outer hair cells (OHC) in the organ of Corti (OC) [3],[4]. The OC is the sensory transducing apparatus in the cochlea and is composed of one row of inner hair cells (IHC) and three rows of OHCs that are separated by two pillar cells (PCs) that form the tunnel of Corti. Each sensory hair cell is associated with an underlying supporting cell (SC). Although there has been progress in understanding mechanisms of hair cell (HC) and SC differentiation [5],[6], the cellular signals that specify the distinct phenotypes of cochlear IHCs and OHCs are not known [7].

Fibroblast growth factor (FGF) signaling has essential functions at several stages of inner ear development. In the embryonic day 9–10 (E9–E10) developing mouse, FGF3, FGF8, and FGF10 are essential for development of the otic vesicle [8]. These ligands signal through FGF receptor (FGFR) 2b in otic epithelium, and mice lacking *Fgfr2b* show impaired otic vesicle development [9]. At later stages of development, FGF signaling is required for morphogenesis of the organ of Corti. At E11.5, *Fgfr1* is expressed in the ventromedial wall of the otocyst, the region that will give rise to the cochlea [10]. At E15, *Fgfr1* expression is observed in the sensory epithelium of the developing cochlea [11],[12]. Conditional disruption of *Fgfr1* in sensory epithelial progenitor cells (with *Foxg1^cre^*) resulted in a severe reduction in HC number, possibly due to reduced proliferation of progenitor cells [10]. A similar phenotype was also observed in organ cultures treated with FGFR inhibitors [11]. *Fgf20* is expressed in the presumptive epithelial domain of the developing cochlea at E13.5 and antibody inhibition of FGF20 in cochlear organ culture resulted in fewer SCs and HCs [11]. These studies suggest that FGF20 might be the ligand for FGFR1 during the early growth and differentiation stages of cochlear development.

At later stages of organ of Corti development (after E15), inhibition of FGF signaling results in loss of PCs, suggesting an additional stage-specific role for FGF signaling [13]. Genetic and gene expression data suggest that this function is mediated by FGF8 signaling to FGFR3. *Fgfr3* is expressed after E15.5 in undifferentiated postmitotic progenitor cells that are thought to have the capacity to form OHCs, Deiters' cells (DCs), PCs, and Hensen's cells (HeCs) [12]–[15]. Genetic disruption of *Fgfr3* prevents the differentiation of PCs and the formation of the tunnel of Corti and results in deafness [13],[15],[16]. FGF8 is expressed in IHCs where it induces differentiation of PCs and formation of one row of OHCs through signaling to FGFR3 [10],[17],[18].

The mechanisms that regulate the formation of OHCs are particularly significant, given the importance of OHCs for hearing function and age-related hearing loss. Although mouse mutants lacking FGFR1 suggest a role for FGF signaling in OHC development [10], the underlying mechanisms regulating OHC development are not known. Here we generated mice lacking FGF20 (*Fgf20^βGal/βGal^*). We show that *Fgf20^βGal/βGal^* mice are viable, healthy, and congenitally deaf, specifically lack OHCs and outer supporting cells, and have patterning defects throughout most of the cochlear sensory epithelium. These studies show that the organ of Corti can be subdivided into developmentally distinct medial (IHCs and inner SCs) and lateral (OHCs and outer SCs) compartments that are under the control of distinct developmental programs. This model posits the existence of distinct progenitor cells that give rise to medial and lateral compartments of the OC.

## Results

### Loss of *Fgf20* Results in Congenital Hearing Loss and Disorganization of the Organ of Corti

**Figure 1:**
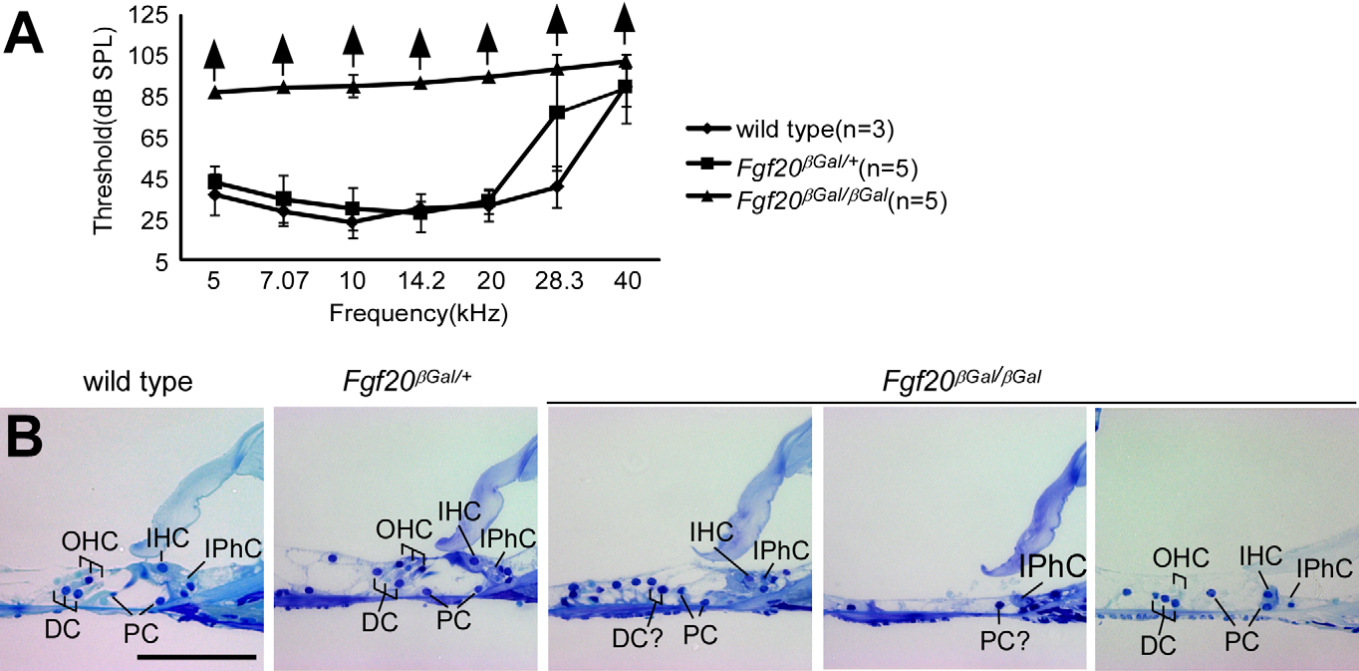
Congenital hearing loss and cochlear dysgenesis caused by loss of *Fgf20* in the developing cochlear sensory epithelium. (A) Auditory brainstem response test of 6-wk-old mice showing normal and comparable thresholds in wild type (*n* = 3) and *Fgf20^βGal/+^* (*n* = 5) mice and a lack of response in *Fgf20^βGal/βGal^* mice (*n* = 5) in the 5–40 kHz range. (B) Histology of the inner ear showing normal organ of Corti structure in wild type and *Fgf20^βGal/+^* cochlea in which there are three outer hair cells (OHC), one inner hair cell (IHC), three Deiters' cells (DC), two pillar cells (PC), and several inner phalangeal cells (IPhC). *Fgf20^βGal/βGal^* cochlea showed disorganization and loss of sensory cells in the organ of Corti. Scale bar: 100 µm.

To study the function of *Fgf20* in vivo, we generated *Fgf20* null mice in which exon 1 was replaced with a β-galactosidase gene (*Fgf20^βGal^*) (Figure S1). Homozygous *Fgf20^βGal/βGal^* mice were viable, fertile, and healthy. However, *Fgf20^βGal/βGal^* mice lacked auditory perception (no ear twitch response to loud noise) and had auditory brainstem response (ABR) thresholds greater than 40 db above controls in the 5–20 kHz range (Figure 1A). Histological sections of the adult inner ear of *Fgf20^βGal/βGal^* mice showed normal gross morphology of the temporal bone and cochlea (Figure S1E–G); however, the OC showed significant dysmorphology, with variability in the degree of disorganization along the length of the cochlea (Figure 1B). Some sections showed almost complete absence of sensory HCs and SCs, while other sections showed loss of OHCs and DCs. In contrast, wild type and heterozygous littermates showed normal cochlear organization, with one IHC, three OHCs, one inner and outer pillar cell (IPC, OPC), and three DCs (Figure 1B).

### *Fgf20* Is Expressed in the Inner Ear Prosensory Epithelium and in Differentiated Supporting Cells

**Figure 2:**
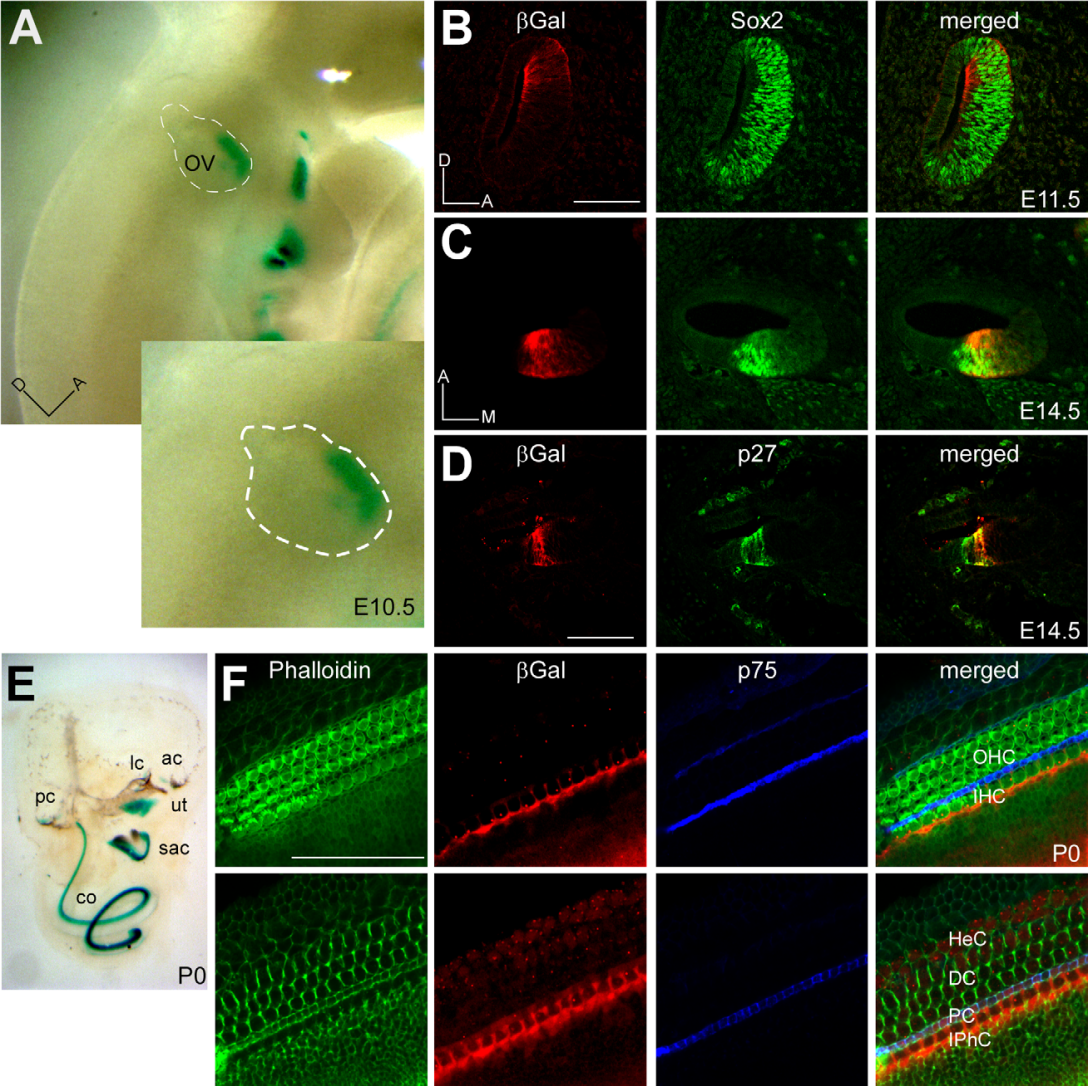
Expression of *Fgf20* in the developing inner ear. (A) βGal staining of E10.5 embryos showing anterio-ventral expression of *Fgf20* in the otic vesicle (OV). (B,C) Co-labeling of βGal and Sox2 showing that at E11.5 (B), the *Fgf20* expression domain is within the Sox2 expressing sensory patch and at E14.5 (C), *Fgf20* expression overlaps with the Sox2 expressing sensory domain. (D) Staining for βGal and for p27 expression showing that at E14.5, *Fgf20* expression partially overlaps with the medial region of p27 expression in the cochlear sensory epithelium. (E) βGal staining at P0 showing *Fgf20^βGal^* expression in all sensory domains of the inner ear, including the cochlea (co), utricle (ut), saccule (sac), and crista of the semicircular canals (pc, lc, ac). (F) Co-staining of βGal, phalloidin, and p75 (pillar cells, PC) in P0 cochlea showing low-level *Fgf20* expression in medial supporting cells (Deiters' cells, DC) and HeC (lower panels), moderate expression in PCs, and highest expression in the inner phallangial cells (IPhC). *Fgf20^βGal^* appears to be excluded from hair cells (upper panels). Scale bar: 100 µm. D, dorsal; A, anterior; M, medial.

To identify spatial and temporal patterns of *Fgf20* expression in the developing inner ear, we stained whole mount preparations for β-galactosidase (*βGal*) activity. In *Fgf20^βGal/+^* embryos, βGal was first detected in the anterio-ventral region of the otic vesicle at E10.5, the region of the otic vesicle where sensory progenitor cells are located (Figure 2A) [19]. In histological sections of the otic vesicle, Fgf20-βGal was expressed within the domain of Sox2+ sensory progenitor cells at E11.5 (Figure 2B). At E14.5, the time of sensory cell specification, Fgf20-βGal was expressed in the Sox2+, p27+ sensory domain (Figure 2C,D), in an apical to basal graded expression pattern, similar to previously reported expression patterns for *Fgf20* [11]. At postnatal day 0 (P0), a time when almost all sensory cells have completed differentiation, Fgf20-βGal was expressed throughout all inner ear sensory epithelia (Figure 2E and Figure S2). In the cochlea, Fgf20-βGal expression was restricted to SCs and was expressed in a graded medial to lateral pattern, with highest levels in the inner phalangeal cells (IPhC) and lower levels in PCs (Figure 2F and Figure S2A). Fgf20-βGal was also expressed in the vestibular sensory organs of the inner ear, including the maculae of the utricle and saccule and cristae of the semicircular canals (Figure S2B–F). *Fgf20^βGal/βGal^* mice did not show any vestibular dysfunction (unpublished data).

### FGF20 Is Required for Patterning Cochlear Sensory Cells and Formation of the Lateral Compartment

**Figure 3:**
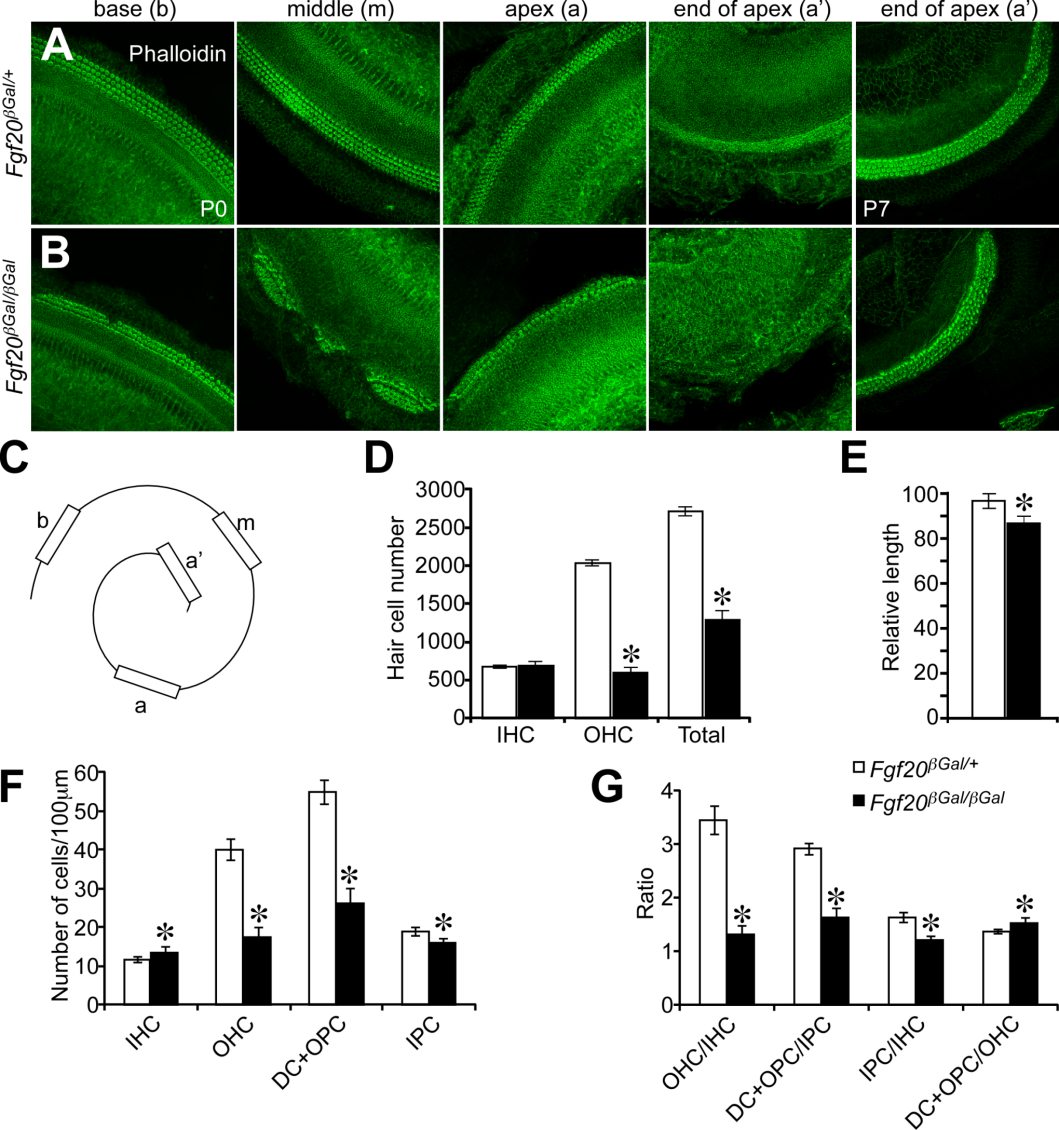
*Fgf20* is necessary for formation of the lateral compartment of the organ of Corti. Phalloidin staining of P0 and P7 cochleae showing hair cells. In *Fgf20^βGal/+^* cochlea, (A) there is one row of inner hair cells (IHC) and three rows of outer hair cells (OHC) throughout the cochlea. At P0, apical hair cells are not fully differentiated; by P7, apical differentiation is complete. In *Fgf20^βGal/βGal^* cochlea (B), the cochlear base (base) has one row of inner hair cells and two rows of outer hair cells. The mid-cochlea (middle) forms patches of hair cells with two rows of IHCs and three rows of OHCs separated by gaps that lack hair cells. The apex (apex) contains hair cells that are much less differentiated and the distal apex (end of apex) contains no identifiable hair cells. At P7, the hair cells in the distal apex of *Fgf20^βGal/βGal^* and *Fgf20^βGal/+^* cochlea are comparable. (C) Diagram showing the cochlear regions examined in (A, B); b, base; m, middle; a, apex, a′, end of apex. (D) Quantification of hair cell numbers at P4. Total numbers of IHCs and OHCs were counted from *Fgf20^βGal/+^* (*n* = 3) and *Fgf20^βGal/βGal^* (*n* = 3) cochlea. The numbers of IHCs were comparable, while the numbers of OHCs were decreased by 70% in *Fgf20^βGal/βGal^* compared to *Fgf20^βGal/+^* cochlea. Total numbers of hair cells were decreased by 50% in *Fgf20^βGal/βGal^* cochlea. (E) Length of cochlea at P4. *Fgf20^βGal/βGal^* cochlea length (*n* = 3) was decreased by 10% compared to *Fgf20^βGal/+^* (*n* = 3). (F, G) Quantification of supporting cell numbers at P0. (F) Number of supporting cells, identified by staining for Prox1 (Figure S3G,H), and hair cells, were counted from base, middle, and apex regions of the OC and normalized to 100 µm from *Fgf20^βGal/+^* (*n* = 6) and *Fgf20^βGal/βGal^* (*n* = 8) mice. IHC numbers were slightly increased while OHC numbers were decreased by 57% in *Fgf20^βGal/βGal^* compared to *Fgf20^βGal/+^* cochlea. Numbers of Deiters' cells (DC) and outer pillar cells (OPC) were decreased by 52%, whereas numbers of inner pillar cells (IPC) were only slightly decreased in *Fgf20^βGal/βGal^* compared to *Fgf20^βGal/+^* cochlea. (G) Ratio of hair cells and supporting cells in P0 cochlea. The ratio of outer compartment cells (OHC, OPC, DC) to inner compartment cells (IHC, IPC) was decreased in *Fgf20^βGal/βGal^* compared to *Fgf20^βGal/+^* cochlea. Small changes in the ratio of outer compartment supporting cells (DC+OPC) to OHCs and inner compartment supporting cells (IPC) to IHCs were observed. * *p*<0.01.

To further analyze the hair cell phenotype in the cochlea, we isolated whole cochleae at P0 and stained with phalloidin, as well as with antibodies against Myo6 and Calretinin (Figure 3 and Figure S3) [20],[21]. Wild type cochleae showed three rows of OHCs and one row of IHCs throughout the OC (Figure 3A). However, in the cochlea of *Fgf20^βGal/βGal^* newborn pups, the proximal base region contained only two rows of OHCs and one row of IHCs. In the middle and apical regions, patches of HCs were observed (Figure 3B). Such patches typically contained three rows of OHCs and two rows of IHCs, and there were no HCs in the regions between the patches. Finally, no distinct phalloidin or Myo6 positive HCs were present in the most apical region. Because HC differentiation progresses from the base to the apex of the cochlea, we sought to determine whether differentiation of HCs in the distal apex region of *Fgf20^βGal/βGal^* cochlea was delayed or whether HCs were lost. At P7, expression of HC markers in the distal apex of *Fgf20^βGal/βGal^* and *Fgf20^βGal/+^* cochlea were comparable (Figure 3 and Figure S3), indicating that HCs were not lost but rather delayed in differentiation. Consistent with delayed HC differentiation, at E16.5, HCs were undifferentiated in the middle region of the cochlea of *Fgf20^βGal/βGal^* compared to *Fgf20^βGal/+^* cochlea (Figure S3A,B).

To identify whether SCs were properly formed, we stained P0 cochlea with antibodies against Prox1 and p75. At P0, Prox1 is expressed at high levels in DCs and PCs, while p75 labels PCs and HeCs [22],[23]. In *Fgf20^βGal/+^* cochleae, there were two rows of PCs, three rows of DCs, and one row of HeCs (Figure S3G). Immunolabeling of *Fgf20^βGal/βGal^* cochleae showed that all the SC types existed, although with dysmorphology. Similar to HC patterns, two rows of DCs and two rows of PCs were formed at the base region of *Fgf20^βGal/βGal^* cochlea (Figure S3H). In the middle region, where HCs were clustered in patches, SCs were formed in accordance with the hair cell pattern (three rows of DCs and two rows of PCs). No SCs were observed in the space between the sensory patches. Interestingly, within a patch, OHCs were surrounded by PCs and HeCs, as indicated by continuous p75 staining (Figure S3J). Unlike HCs, apical SCs were differentiated, as indicated by Prox1 staining (Figure S3G,H), suggesting that apical SCs may develop normally in the absence of FGF20.

Because of the delay in HC differentiation and patch formation, the total numbers of cochlear HCs were quantified at P4, a time when both *Fgf20^βGal/+^* and *Fgf20^βGal/βGal^* cochlea appeared fully differentiated (Figure S3K). Surprisingly, although there were two rows of IHCs in patches and no IHCs in the region between patches, the total number of IHCs was the same in *Fgf20^βGal/+^* and *Fgf20^βGal/βGal^* cochlea (680±18, *n* = 4 in *Fgf20^βGal/+^* and 685±58, *n* = 3 in *Fgf20^βGal/βGal^*, *p* = 0.4). However, the number of OHCs was decreased by 70% in *Fgf20^βGal/βGal^* compared to *Fgf20^βGal/+^* cochlea (2035±42 in *Fgf20^βGal/+^* and 601±60 in *Fgf20^βGal/βGal^*, *p*<0.001) (Figure 3D). In addition, cochlear length was decreased by 10% in *Fgf20^βGal/βGal^* compared to *Fgf20^βGal/+^* mice (Figure 3E). We also counted the number of SCs (DCs, OPCs, and IPCs) normalized to 100 µm intervals. Similar to the large decrease in the number of OHCs, the number of DCs+OPCs was decreased by 52% in *Fgf20^βGal/βGal^* compared to *Fgf20^βGal/+^* cochlea (55±3, *n* = 6 in *Fgf20^βGal/+^* and 26±4, *n* = 8 in *Fgf20^βGal/βGal^* per 100 µm, *p*<0.001) (Figure 3F). In contrast, the number of IPCs was decreased by only 15% in *Fgf20^βGal/βGal^* compared to *Fgf20^βGal/+^* cochlea (19±1 in *Fgf20^βGal/+^* and 16±1 in *Fgf20^βGal/βGal^* per 100 µm, *p*<0.01) (Figure 3F). Next, we compared the ratio of different cell types in *Fgf20^βGal/βGal^* and *Fgf20^βGal/+^* cochlea. In *Fgf20^βGal/+^* cochlea, the ratio of OHC/IHC was 3.4±0.3. However, in *Fgf20^βGal/βGal^* cochlea this ratio was decreased (by 62%) to 1.3±0.6 (*p*<0.001). Additionally, the ratio of DC+OPC/IPC in *Fgf20^βGal/+^* cochlea was 2.9±0.1, but was decreased (by 45%) to 1.6±0.2 in *Fgf20^βGal/βGal^* cochlea (*p*<0.001). Interestingly, within the lateral and medial compartments, the ratio of DC+OPC/OHC (1.4±0.0 in *Fgf20^βGal/+^* and 1.5±0.1 in *Fgf20^βGal/βGal^*, *p*<0.01) and IPC/IHC (1.6±0.1 in *Fgf20^βGal/+^* and 1.2±0.1 in *Fgf20^βGal/βGal^*, *p*<0.001) was slightly increased (by 7%) or decreased (by 25%), respectively, in *Fgf20^βGal/βGal^* compared to *Fgf20^βGal/+^* cochlea. These ratios indicate that absence of FGF20 primarily affects lateral compartment cells (i.e., OHCs and DCs).

### Exogenous FGF Rescues Loss of Sensory Cell Before E14.5

**Figure 4:**
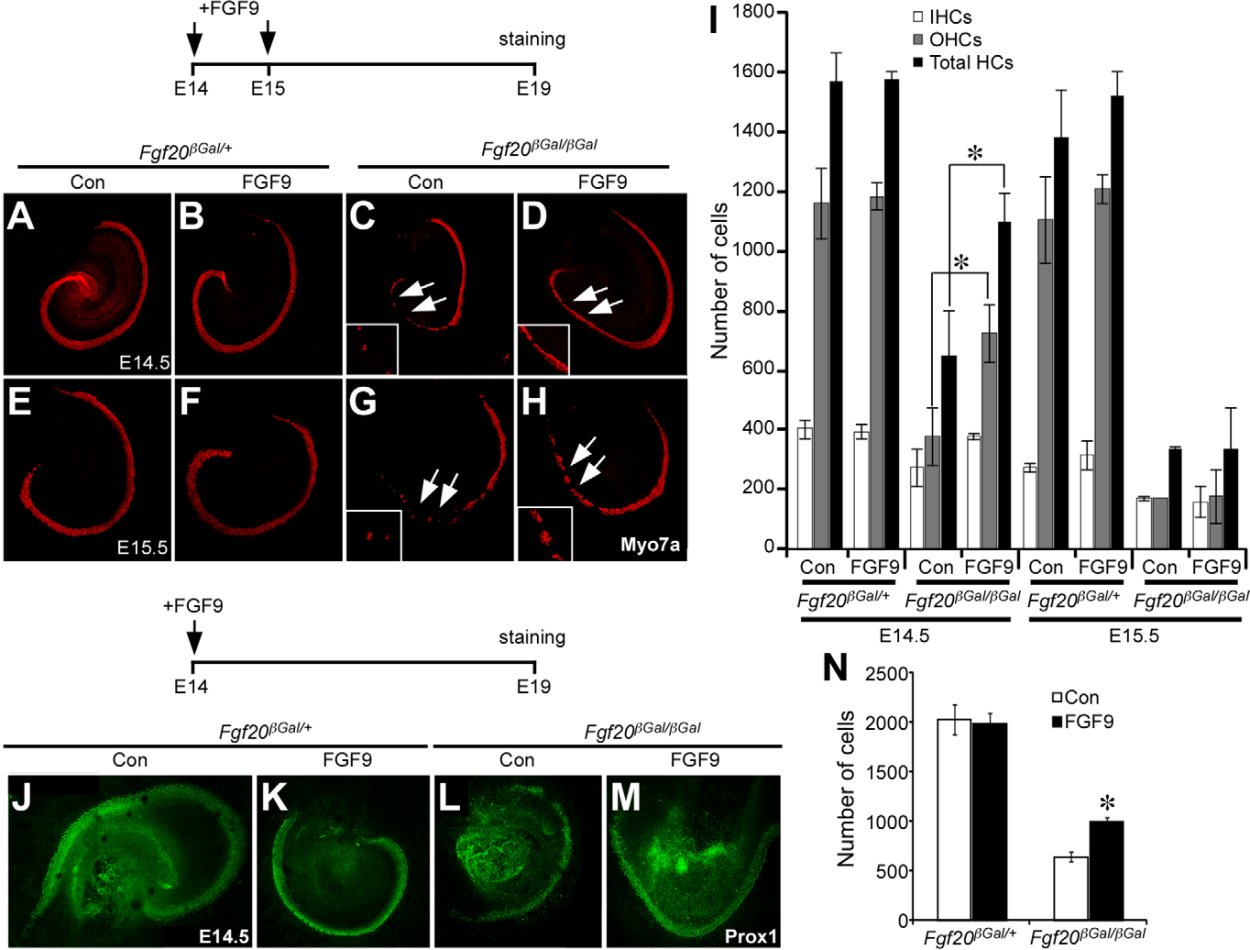
FGF20 initiates lateral compartment differentiation before E14.5. (A–H) Immunostaining of Myo7a in *Fgf20^βGal/+^* and *Fgf20^βGal/βGal^* cochlear explants treated with or without FGF9 and cultured for 5 d (schematic). Treatment of *Fgf20^βGal/+^* explants with FGF9, either at E14.5 (B) or E15.5 (F), did not have any effect on hair cell number compared to untreated explants (A,E). Treatment of *Fgf20^βGal/βGal^* explants with FGF9 at E14.5 resulted in increased numbers of hair cells and decreased gaps (arrows) between hair cell clusters (D) compared to untreated explants (C). Treatment of *Fgf20^βGal/+^* or *Fgf20^βGal/βGal^* explants with FGF9 at E15.5 did not affect hair cell number or the formation of gaps (arrows) lacking sensory cells (G, H). (I) Quantitation of the number of hair cells. The number of OHCs and total hair cells were rescued by treatment with FGF9 at E14.5 but not at E15.5. * *p*<0.05. (J–M) Immunostaining for Prox1 in *Fgf20^βGal/+^* and *Fgf20^βGal/βGal^* explants treated with or without FGF9 at E14.5 and cultured for 5 d (schematic). Treatment of *Fgf20^βGal/+^* explants with FGF9 did not affect supporting cell number (K) compared to untreated explants (J). Treatment of *Fgf20^βGal/βGal^* explants with FGF9 at E14.5 resulted in increased numbers of supporting cells and decreased gaps between sensory cell clusters (M) compared to untreated explants (L). (N) Quantitation of numbers of supporting cells in explants. The number of supporting cells was partially rescued in *Fgf20^βGal/βGal^* explants by treatment with FGF9 at E14.5.

Next we asked whether loss of the lateral compartment was due to loss of sensory domain progenitor cells. To do this, we labeled E13.5 or E14.5 cochlea for Sox2, a marker for sensory progenitors, or Jag1, a marker for Kölliker's organ [24],[25]. The expression pattern of Sox2 and Jag1 was comparable in *Fgf20^βGal/βGal^* and *Fgf20^βGal/+^* cochlea at E13.5 and E14.5 (Figure S4A–F). Additionally, cell proliferation was comparable in *Fgf20^βGal/βGal^* and *Fgf20^βGal/+^* E13.5 cochlea (Figure S4G,H), indicating that the sensory domain had formed normally. Next, we hypothesized that FGF20 may play a role in lateral compartment differentiation. To test this, we isolated E13.5 or E14.5 cochlea and treated explants with 1 µM FGF9 (which shows similar biochemical activity in vitro compared to FGF20) [26] beginning at E13.5, E14.5, E15.5, and E16.5. Control cultures were maintained in parallel, but did not receive FGF9. Cochlear explants were cultured in these media for 5 d and then stained with Myo7a antibodies (to identify HCs) [20],[27]. Untreated *Fgf20^βGal/+^* explants showed normal patterning, with one row of IHCs and 3–4 rows of OHCs (Figure 4A,E and Figure S5A,E). Also, untreated *Fgf20^βGal/βGal^* explants showed the expected patterning defects (patches of HCs and SCs towards the apical cochlea) and loss of HCs (Figure 4C,G and Figure S5C,G). Notably, however, treatment of *Fgf20^βGal/βGal^* explants with FGF9 at E13.5 and E14.5 resulted in rescue of the cochlear phenotype, with the cochlea showing a normal and contiguous pattern of sensory cells and increased numbers of OHCs (162±53, *n* = 2 without FGF9 and 486±122, *n* = 3 with FGF9 treatment at E13.5, *p*<0.05, and 376±96, *n* = 3 without FGF9 and 725±100, *n* = 3 with FGF9 treatment at E14.5, *p*<0.01) compared to untreated explants (Figure 4C,D,I and Figure S5C,D,I). Finally, treatment of *Fgf20^βGal/βGal^* explants with FGF9 at E15.5 or E16.5 did not affect the number of HCs (168±0, *n* = 2 without FGF9 and 176±90, *n* = 3 with FGF9 treatment at E15.5, *p* = 0.9, and 238±40, *n* = 4 without FGF9 and 279±47, *n* = 4 with FGF9 treatment at E16.5, *p* = 0.3) (Figure 4G,H,I and Figure S5G,H,I), indicating that normal differentiation of lateral compartment cells requires active FGF signaling prior to E14.5. Patterning and numbers of SCs were also rescued by FGF9 treatment (640±49, *n* = 3 without FGF9 and 1001±39, *n* = 4 with FGF9 treatment at E14.5, *p*<0.001) (Figure 4J–N). BrdU labeling of these cultures indicated that treatment of *Fgf20^βGal/βGal^* cochlea with FGF9 did not induce renewed proliferation within the sensory epithelia (Figure S5J and K) at this stage, which indicated that FGF9 treatment functioned to induce lateral compartment cell differentiation into HCs and SCs. Also, treatment of *Fgf20^βGal/+^* explants with FGF9 did not change the morphology or number of HCs or SCs, indicating that FGF signaling is not sufficient to induce ectopic HC or SC formation (Figure 4B,F,I,K and Figure S5B,F,I). Similar experiments were repeated with recombinant FGF20 protein with qualitatively similar results, although FGF20 was less active than FGF9 in this assay (unpublished data).

### The Region between Patches in *Fgf20^βGal/βGal^* Cochlea Contains Undifferentiated Postmitotic Sensory Cells

**Figure 5:**
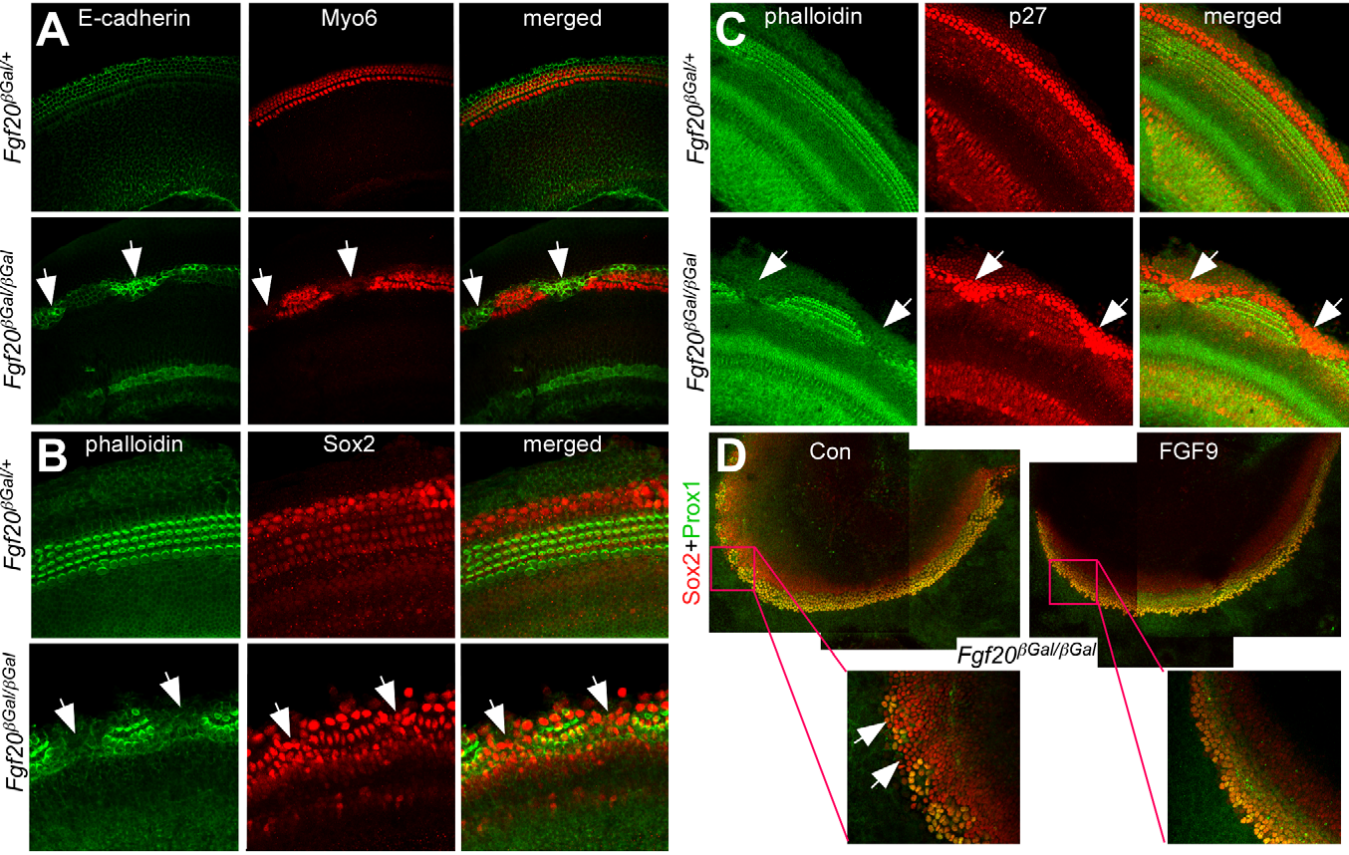
Lack of FGF20 results in undifferentiated lateral compartment cells. (A) Co-immunostaining of E-Cadherin and Myo6 showing that E-Cadherin stains lateral compartment cells in *Fgf20^βGal/+^* cochlea (A, upper). In *Fgf20^βGal/βGal^* cochlea, E-Cadherin stains all lateral compartment cells including cells localized in the region between sensory patches (A, lower, arrow). (B) Co-immunostaining of Sox2 and phalloidin showing that Sox2 labels supporting cells in *Fgf20^βGal/+^* cochlea (B, upper). In *Fgf20^βGal/βGal^* cochlea, the region between patches was marked by strong Sox2 staining (B, lower, arrows). (C) Co-immunostaining of p27 and phalloidin showing that p27 stains supporting cells in *Fgf20^βGal/+^*cochlea (C, upper). In *Fgf20^βGal/βGal^* cochlea, the region between patches was marked by strong p27 staining (C, lower, arrows). (D) Co-immunostaining of Sox2 and Prox1 of *Fgf20^βGal/βGal^* explants treated with or without FGF9. Without FGF9 treatment (left), Sox2+/Prox1− cells were localized in the region between sensory patches (arrows). Treatment of *Fgf20^βGal/βGal^* explants with FGF9 induced Sox2+/Prox1− cells to express Prox1 (right).

To determine whether the missing outer compartment cells were lost or were still present in an undifferentiated state, we stained whole cochleae of P0 pups with E-Cadherin antibodies, which marks lateral compartment cells at late gestational and postnatal stages of development [6]. In *Fgf20^βGal/+^* cochlea, E-Cadherin labeled all lateral compartment cells including OHCs, DCs, and HeCs (Figure 5A, upper). Interestingly, in *Fgf20^βGal/βGal^* cochlea, E-Cadherin was highly expressed in the region between the sensory patches where there were no HCs or SCs (Figure 5A, lower), identifying these as potential lateral compartment cells. We also labeled specimens for Sox2 and with phalloidin. At P0, Sox2 labels all supporting cells, but at earlier stages of sensory domain formation (E14.5), Sox2 labels undifferentiated sensory cells [24]. We observed normal patterns of Sox2 expression in *Fgf20^βGal/+^* cochlea. However, in *Fgf20^βGal/βGal^* cochlea, Sox2 was expressed both in supporting cells and in the regions between the sensory patches (Figure 5B arrows). We also examined the expression of p27, a marker of SCs and undifferentiated sensory progenitors [28]. The expression pattern of p27 was similar to that of Sox2, with high expression in cells in the region between the sensory patches (Figure 5C). Although the identity of the cells within these gaps in the sensory epithelium is not known, these expression studies suggest that these cells may be an arrested progenitor-like cell or a differentiated non-sensory cell. To determine whether the lineage precursors of these cells could be rescued, E14.5 *Fgf20^βGal/βGal^* cochlea explants were treated with or without FGF9 and co-stained for Sox2 and Prox1 expression after 5 d in culture. In explants not exposed to FGF9, the region between the patches was Sox2+; Prox1− (Figure 5D, left). However, following exposure to FGF9, these cells became Sox2+; Prox1+ (Figure 5D, right), indicating that the lineage precursors of these cells are undifferentiated sensory cells and that exposure to FGF9 induced their differentiation into lateral HCs and SCs, such as OHCs and DCs. This finding indicates that FGF signaling is required to induce differentiation of cells in the lateral cochlear compartment.

### Undifferentiated Sensory Cells Are Not Influenced by Notch Mediated Lateral Inhibition

**Figure 6:**
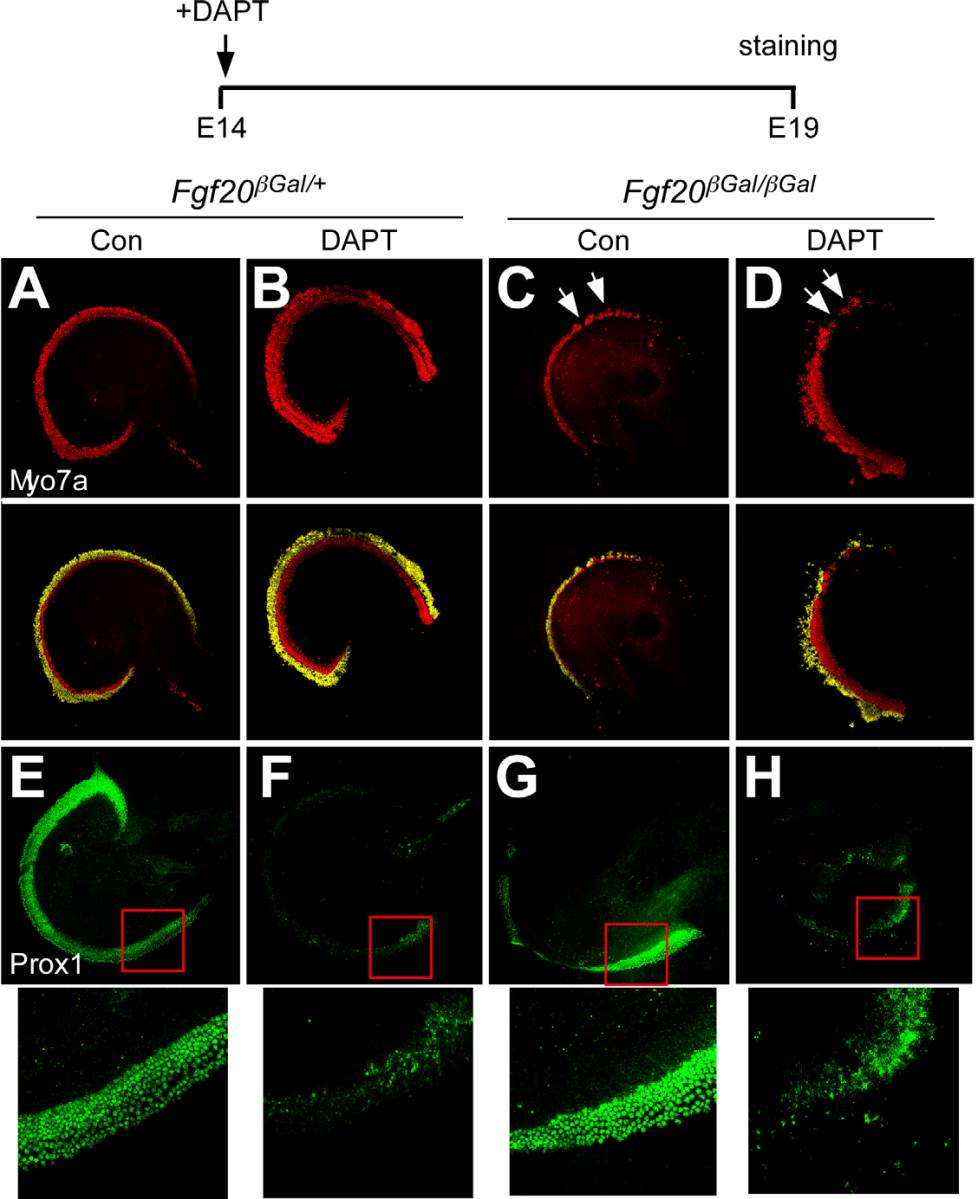
Notch signaling does not influence undifferentiated lateral compartment cells. (A–D) Immunostaining of Myo7a in *Fgf20^βGal/+^* and *Fgf20^βGal/βGal^* explants treated with or without DAPT at E14 and cultured for 5 d (schematic). Treatment of *Fgf20^βGal/+^* explants with DAPT resulted in increased numbers of inner and outer hair cells (B) compared to untreated explants (A). *Fgf20^βGal/βGal^* explants treated with DAPT showed a comparable increase of IHCs (C) but a decreased expansion of the OHC compartment (D), compared to *Fgf20^βGal/+^* explants treated with DAPT (A, B). Lower panels in (A–D) show OHCs false-colored in yellow. (E–H) Staining for Prox1 expression shows absence of Prox1 expressing cells in explants treated with DAPT indicating that inhibition of Notch signaling blocks formation of supporting cells in both *Fgf20^βGal/+^* and *Fgf20^βGal/βGal^* explants. Lower panels show enlarged region (box) with only background Prox1 staining in DAPT treated explants.

**Figure 7:**
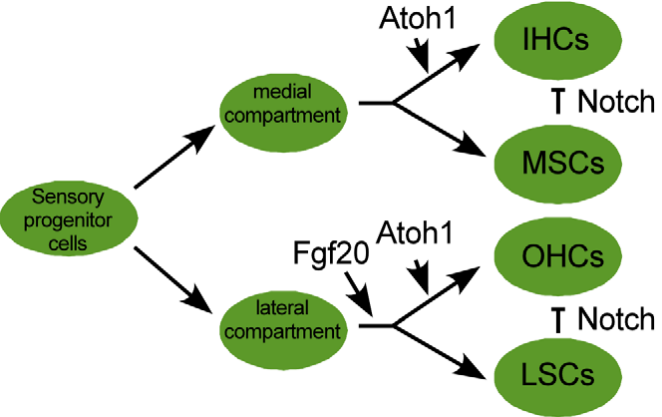
Schematic model of sensory cell development in the organ of Corti. Diagram showing that FGF20 specifically functions to initiate lateral compartment development. The differential activity of FGF20 suggests that there may be separate progenitor cells for the medial and lateral cochlear compartments.

Next, we asked whether FGF20 functions to induce differentiation of a specific cell phenotype in the lateral compartment versus functioning as a gate to permit lateral compartment differentiation. To answer this question, we treated E14.5 cochlea explants with DAPT, a γ-secretase inhibitor, which inhibits the Notch signaling pathway [29]. At this stage of development, the Notch pathway functions to prevent SC differentiation into HCs [30]. In *Fgf20^βGal/+^* explants treated with DAPT, the domain of IHCs and OHCs expanded at the expense of SCs, compared to untreated explants (Figure 6A and B). In *Fgf20^βGal/βGal^* explants, treatment with DAPT also expanded the IHC domain, similar to heterozygous explants treated with DAPT. However, the domain of OHCs in DAPT-treated *Fgf20^βGal/βGal^* explants was still smaller than the OHC domain of DAPT-treated *Fgf20^βGal/+^* explants and also contained patches of undifferentiated sensory progenitor cells (Figure 6C and D). This finding indicates that DAPT treatment did not induce differentiation of otherwise FGF20-dependent precursor cells. Also, we observed a dramatic reduction of SCs following DAPT treatment of either genotype, indicating that all SCs were converted into HCs (Figure 6F and H). Together, these data support a model in which FGF20 functions as a permissive factor that is required to initiate lateral compartment differentiation before E14.5 (Figure 7). Without FGF20, lateral sensory cells remained in an undifferentiated state.

## Discussion

The mechanisms that differentially regulate formation of inner versus outer hair cells are poorly understood. In this study, we show that *Fgf20^βGal/βGal^* mice have a specific deficiency in the formation of OHCs and outer supporting cells that make up the lateral compartment of the organ of Corti. These observations suggest that FGF signaling may regulate the growth or differentiation capacity of a progenitor cell that gives rise to lateral compartment cells and that medial (IHCs and inner SCs) and lateral compartment development may be controlled by distinct mechanisms (Figure 7). Additionally, since *Fgf20^βGal/βGal^* mice are viable and healthy, but are congenitally deaf, *FGF20* is likely a candidate gene for hereditary deafness in humans. Interestingly, the *FGF20* gene is located on human chromosome 8p22–21.3 within the autosomal recessive non-syndromic hearing impairment locus, DFNB71 [31].

### Timing of FGF Signaling and Sensory Cell Differentiation

To identify the developmental time when FGF20 functions to regulate lateral compartment differentiation, rescue experiments were performed in which *Fgf20^βGal/βGal^* cochlea were placed in culture prior to differentiation (E13.5) and then FGF9 was added to the culture at different time points. These experiments showed that the lateral compartment differentiation defects in *Fgf20^βGal/βGal^*mice could only be rescued if FGF9 was added at or before E14.5. However, treatment with FGF9 at or after E15.5 failed to rescue the phenotype of *Fgf20^βGal/βGal^* mice. This is interesting because E14.5–15.5 corresponds to the time when sensory cell specification is completed and HC and SC differentiation begins [5],[32].

The changes in the cochlear epithelium that renders it non-responsive to FGF signaling after E14.5 are not known. Possibilities include loss of FGFR1 expression, uncoupling of FGFR1 to cellular signal transduction pathways, or loss of cofactors required for ligand activation of FGFR1. In lung development, a feed forward signaling loop couples FGF9 with Wnt/β-catenin signaling and maintenance of FGFR expression. Loss of *Fgf9* resulted in loss of *Fgfr1* and *Fgfr2* expression and subsequent loss of responsiveness of explanted lung to exogenous FGF9 [33]. If a similar feed forward loop functions in the inner ear prosensory epithelium, loss of FGFR expression in *Fgf20^βGal/βGal^* mice could explain the loss of responsiveness to exogenous FGF after E14.5. However, in the inner ear, FGF20 continues to be expressed in IPhCs and at low levels in PCs until early postnatal ages (Figure 2). This suggests that FGF20 signaling may have additional roles in cochlear development. At P0, βGal staining indicates that *Fgf20* is expressed at highest levels in the cochlear apex (Figure 2). Since differentiation of the apical cochlea is delayed in *Fgf20^βGal/βGal^* mice (Figure 3), FGF20 may function at later stages of development to promote sensory cell maturation.

Because damage or loss of OHCs is thought to be a major cause of sensorineural hearing loss, efforts to restore hearing in some patients with sensorineural hearing loss will require regeneration of OHCs. Understanding the changes that occur in sensory progenitor cells between E14.5 and E15.5 is important because they may provide clues about pathways required for reactivation of OHC progenitors in the adult or protecting OHCs from ototoxic damage. Although FGF20 signaling alone may not be sufficient to induce regeneration of OHCs, it may be required in combination with other signaling molecules. For example, in lung development, responsiveness of lung tissue lacking FGF9 can be restored by simultaneously treating *Fgf9* ^−*/*−^ explants with activators of Wnt/β-catenin signaling and with FGF9 [34].

### The FGF9 Family Has Unique Signaling Properties in Development

The *Fgf9* subfamily includes *Fgf16* and *Fgf20* [35]. Consistent with the conserved sequences within this subfamily, the biochemical activities of FGF20 are similar to that of FGF9 and FGF16 [26]. In vitro, FGF20 binds and activates c splice variants of FGFR1, FGFR2, and FGFR3, which are generally expressed in mesenchymal cells, and b splice variants of FGFR3, which are expressed in epithelial cells [26]. However, the phenotype of *Fgf20^βGal/βGal^* mice is most similar to that of *Fgfr1* conditional deletion mutants, in which epithelial *Fgfr1* was inactivated in the developing inner ear sensory epithelium with *Foxg1^cre^* [10]. The phenotypic similarities strongly suggest that FGFR1 is the physiological receptor for FGF20. Because, in vitro, FGF20 activates FGFR1c to a much greater extent than FGFR1b [26], the FGFR1c variant may be expressed in the developing cochlear sensory epithelium. Alternatively, unique cofactors within the cochlear sensory epithelium may allow FGF20 to activate FGFR1c.

### FGF Signaling and Hair Cell Regeneration

The sensory epithelium of the mammalian cochlea cannot regenerate following ototoxic or noise damage; however, the avian and amphibian inner ear responds to ototoxic or noise-induced injury with a robust regenerative response that results in complete functional recovery [36]. The underlying mechanisms accounting for this difference in regenerative capacity are not understood. However, in principal, therapeutic reactivation of appropriate signaling pathways in the mammalian inner ear should be able to recapitulate the avian response, resulting in both functional repair and prevention of further pathology. Our observation that FGF20 functions as a permissive factor for lateral compartment differentiation suggests that FGF signaling may be a necessary factor for promoting inner ear regeneration. Additionally, zebrafish lacking FGF20 are viable and healthy, but have defects in their ability to regenerate damaged fins [37]. These observations suggest that FGF signaling, and specifically FGF20 or related FGFs, may be important factors for regeneration of a variety of tissues, including the inner ear. Inducible genetic systems in the mouse and the identification of signaling pathways that interact with FGF20 will be required to test the protective or regenerative potential of FGF20 in noise or ototoxic damaged mammalian inner ear.

## Materials and Methods

### Ethics Statement

This study was carried out in strict accordance with the recommendations in the Guide for the Care and Use of Laboratory Animals of the National Institutes of Health. The protocol was approved by the Washington University Division of Comparative Medicine Animal Studies Committee (Protocol Number 20100223). All efforts were made to minimize animal suffering.

### Generation of *Fgf20* Mutant Mice

The *Fgf20* targeting construct was made using recombineering methods as previously reported [38]. Briefly, exon1 of *Fgf20* was replaced with a βGal–LoxP-neomycin-LoxP cassette to generate *Fgf20^βGal(neo)/+^* mice. The *neomycin* gene was eliminated by mating with *β-actin^cre^* mice to generate *Fgf20^βGal/+^* mice. *Fgf20^βGal/+^* males and females were crossed to generate *Fgf20^βGal/βGal^* mice. Genotyping was performed using PCR1: CTGCATTC GCCTCGCCACCCTTGCTACACT; PCR2: GGATCTGCAGGTGGAAGCCGGTGCGGCAGT; PCR3: GGCCTTCCTGTAGCCAGCTTTCATCAACAT primers, which amplify wild type (335 bp) and mutant (498 bp) PCR fragments as indicated in Figure S1A. Mice were maintained on a 129X1/SvJ;C57B6/J mixed background. *Fgf20^βGal/+^* and *Fgf20^βGal/βGal^* mice were viable and fertile.

### Auditory Brainstem Response Test

Mice were anesthetized by i.p. administration of ketamine (80 mg/kg) and xylazine (15 mg/kg), and maintained at 37°C throughout the testing. ABR testing was carried out in a single walled sound-attenuating room. Testing was similar to what has been previously described [39]. Briefly, stimulus presentation and data acquisition were performed with TDT System 3 equipment using SigGen and BioSig software (Tucker Davis Technologies). An ES-1 electrostatic speaker was placed 7 cm from the animal's right ear. Toneburst stimuli (5, 10, 20, 28, and 40 kHz) were 5 ms in length with a 1-ms rise/fall time. Stimuli were presented at decreasing intensities in 5 dB steps until Wave I was no longer observed. Auditory profiles were recorded using platinum subdermal needle electrodes (Grass Technologies) placed with the recording electrode behind the right pinna, the reference electrode at the vertex, and ground electrode in the skin of the back. Responses were amplified and filtered (X 100,000 and low filter: 100 Hz, high filter: 3,000 Hz) using a Grass P 55 preamp. Tonebursts at each frequency and intensity were presented 1,000 times. Stimulus levels were calibrated using SigCal (Tucker Davis Technologies) program with an ACO Pacific ¼ inch microphone placed where the mouse's ear would be located.

### RNA Extraction, cDNA Synthesis, and Quantitative RT-PCR

For detection of *Fgf20* mRNA, E14.5 embryos were dissected and inner ear tissue was isolated. RNA was extracted with the RNeasy kit (Qiagen). cDNA synthesis used the SuperScript III First-Strand Synthesis System for reverse transcription–polymerase chain reaction (Invitrogen), following the manufacturer's protocol. Mouse *Fgf20* mRNA levels were quantified using a *Taq*Man gene expression assay (ABI Mm00748347_m1). *Taq*Man assays were run in an ABI7500 fast real-time PCR machine.

### βGal Staining

Cochleae were dissected from P0 pups in PBS and fixed overnight in Mirsky's Fixative (National Diagnostics). For whole mount staining, samples were washed three times in PBT (PBS, 0.1% Tween-20) and incubated in βGal staining solution (2 mM MgCl_2_, 35 mM potassium ferrocyanide, 35 mM potassium ferricyanide, 1 mg/mg X-Gal in PBT) at 37°C until color reaction was apparent. Samples were washed in PBS fixed in 10% formalin and imaged under a dissecting microscope. For staining histological sections, samples were cryosectioned, washed with PBS, and incubated in βGal staining solution. Samples were washed in PBS, embedded, and photographed.

### Histology

For adult histology, mice were sacrificed with an overdose of pentobarbital (200 mg/kg). Temporal bones were dissected free from the skull and broken in half to expose the cochlea, which was perfused via the round window with a solution containing 4% paraformaldehyde and 0.1% glutaraldehyde. Cochleae were further fixed in this solution overnight and then rinsed free of aldehyde with several changes of PBS. They were then post-fixed in 1% osmium tetroxide (30 min) and rinsed and dehydrated through a series of acetones. Tissues were then infiltrated and embedded in an epon-araldite mixture and polymerized overnight at 60°C. Ten adjacent, 4 µm thick sections were saved from the midmodiolar plane from each cochlea, counterstained with toluidine blue, and coverslipped.

For frozen sections, embryos were fixed with 4% paraformaldehyde overnight and washed with PBS. Samples were soaked in 30% sucrose and embedded in OCT compound (Tissue-Tek). Samples were sectioned (12 µm) and stored at −80°C for immunohistochemistry.

### Hair Cell and Supporting Cell Counting

For total hair cell counting, P4 cochleae stained with phallodin were used because both *Fgf20^βGal/+^* and *Fgf20^βGal/βGal^* cochleae were completely differentiated at this stage. For supporting cell counting, P0 cochleae stained with either phallodin or Prox1 were used. Because of the incomplete staining pattern of Prox1 from the base to the apex, we could not count all of the supporting cells. Instead, we counted more than 300 µm regions of the base, middle, and apex of the cochlea and normalized counts to 100 µm. Inner and outer hair cells were identified by location and morphology of phalloidin staining. Inner pillar cells were distinguished by location and morphology among Prox1+ cells. Deiters' cells and outer pillar cells were counted by exclusion of inner pillar cells from Prox1+ cells. Cell counting was performed using Image J software.

### Organotypic Explant Cochlear Cultures

Embryonic mouse cochlear cultures were established as described previously [40] with minor modifications. In brief, cochleae from *Fgf20^βGal/+^* and *Fgf20^βGal/βGal^* embryos at various ages (E13.5–E16.5) were dissected, to expose the sensory epithelium, in ice-cold M199 Hanks solution, transferred to a Ma-Tek dish (Ma-Tek Corporation), coated with Matrigel (BD Biosciences), and maintained at 37°C in vitro in experimental (FGF9 or DAPT) or control culture media for 3–6 d. Recombinant FGF9 and FGF20 protein was obtained from PeproTech Inc. DAPT was obtained from Sigma. To activate FGF signaling, FGF9 culture media (1 µg/ml FGF9+1 mM Heparin in MEM+10% FBS) was added to explant cultures at E13.5, E14.5, E15.5, or E16.5 for 6, 5, 4, or 3 d, respectively (until age of E19.5). Control culture media contained (1 µg/ml heparin in MEM+10% FBS). To inhibit Notch signaling, DAPT (N-[(3,5-Difluorophenyl)acetyl]-L-alanyl-2-phenyl]glycine-1,1-dimethylethyl ester) culture media was added to explant cultures at E14.5 for 5 d (E19.5). DAPT media: 10 µM DAPT (reconstituted in DMSO) in MEM+10% FBS. Control culture media contained DMSO in MEM+10% FBS. Cochleae were treated in pairs (i.e., cochlea from left ear received experimental media while the cochlea from the right ear of the same embryo received control media). The appropriate culture media was replaced every 24 h for all explants. Following incubation, explants were fixed in 4% paraformaldehyde for 30 min and analyzed by immunohistochemistry.

### Immunohistochemistry

Immunohistochemistry was described previously [41]. Briefly, for whole mount immunofluorescence, cochleae were isolated and fixed in 4% PFA overnight at 4°C. Samples were washed with PBS and blocked with PBS containing 0.1% triton X-100 and 0.5% goat serum. Primary antibody was incubated overnight at 4°C. Samples were washed with PBS and incubated with a secondary antibody for 2 h at room temperature. Samples were washed, placed on a glass microscope slide, coverslipped, and photographed using a Zeiss LSM 700 confocal microscope. For section immunofluorescence, frozen sections (12 µm) were washed with PBS and blocked with 0.1% triton X-100 and 0.5% donkey serum. Sections were incubated with primary antibodies in a humidified chamber overnight at 4°C. Sections were then washed and incubated with secondary antibody for 1 h at room temperature. Samples were washed, coverslipped with Vectashield Mounting Media (Vector lab), and photographed using a Zeiss LSM 700 confocal microscope. Primary antibodies used: Phallodin (R&D Systems, 1∶40), Myo6 (Proteus Biosciences, 1∶500), Myo7a (Proteus Biosciences, 1∶500), Calretinin (Millipore, 1∶500), Prox1 (Covance, 1∶500), p27 (Neomarkers, 1∶500), p75 (Chemicon, 1∶500), β-Galactosidase (Abcam, 1∶500), Sox2 (Millipore, 1∶500, Santa Cruz 1∶200), BrdU (BD Biosciences, 1∶500), E-cadherin (Invitrogen, 1∶500), and Jag1 (Santa Cruz 1∶200).

### Statistics

Number of samples is indicated for each experiment. All data are presented as mean ± standard deviation (sd). The *p* value for difference between samples was calculated using a two-tailed Student's *t* test. *p*<0.05 was considered as significant.

## Supporting Information

Figure S1 *Fgf20* gene targeting and morphology of the adult cochlea. (A) Exon1 of the *Fgf20* gene was replaced with a β-galactosidase gene and a PGK promoter-neomycin gene flanked by LoxP recombination sites. In vivo Cre mediated recombination (*β-actin^cre^*) was used to excise the neomycin cassette. (B) Southern blot of wild type, *Fgf20^βGal/+^*, and *Fgf20^βGal/βGal^* mouse DNA digested with BamH1 and probed with a 5′ probe that is extrinsic to the targeting vector. Wild type 15 Kb and mutant 10 Kb bands are indicated. (C) PCR genotyping of the *Fgf20^βGal^* alleles showing wild type (335 bp) and mutant (498 bp) PCR fragments. Orientation of PCR primers is indicated. (D) Quantitative RT-PCR of E14.5 inner ear tissue showing expression of *Fgf20* mRNA in wild type tissue, reduced expression in *Fgf20^βGal/+^* tissue, and no detectable expression in *Fgf20^βGal/βGal^* tissue. (E–G) Thin sections stained with toluidine blue showing comparable cochlear morphology of 2-mo-old wild type (E), *Fgf20^βGal/+^* (F), and *Fgf20^βGal/βGal^*(G) mice.
(TIF)

Figure S2 Expression of *Fgf20* in the developing inner ear. (A–E) Whole mount (B, C) and sections (A,D–F) showing βGal expression in the sensory epithelium of the organ of Corti (A), utricle (B, D), saccule (C, E), and cristae of the semicircular canals (F) at P0.
(TIF)

Figure S3 Hair cell and supporting cell formation in the mouse cochlea. (A,B) Staining of the cochlea with Myo6 expression, showing fewer differentiated hair cells towards the cochlear apex in *Fgf20^βGal/+^* E16.5 embryos (A). In *Fgf20^βGal/βGal^* embryos, no distinctive phalloidin stained or Myo6 expressing hair cells were formed in the apical cochlea at E16.5 (B). (C, D) Staining of the P0 cochlea for Myo6 expression showing staining throughout the length of the cochlea in *Fgf20^βGal/+^* embryos (C). *Fgf20^βGal/βGal^* embryos showed decreased Myo6 staining in the cochlear apex (D). (E, F) Staining of the P7 cochlea for Calretinin expression showing comparable expression levels in *Fgf20^βGal/+^* (E) and *Fgf20^βGal/βGal^* (F) cochleae. (G, H) Staining of the cochlea for Prox1 expression showing two rows of pillar cells and three rows of Deiters' cells throughout the cochlea of *Fgf20^βGal/+^* embryos (G). *Fgf20^βGal/βGal^* embryos had two rows of pillar cells and two rows of Deiters' cells in the base, patches of differentiated supporting cells containing two rows of pillar cells and three rows of Deiters' cells in the middle, and differentiated supporting cells in the apex (H). (I, J) Staining of the cochlea for p75 expression showing differentiated pillar cells (strong staining) and Henson's cells (weak staining) throughout the length of the cochlea of *Fgf20^βGal/+^* embryos (I). Pillar cells and Henson's cells were also identified in *Fgf20^βGal/βGal^* cochlea, but the pattern matched that of hair cells, showing patches of differentiated cells and gaps of unlabeled cells in the middle region of the cochlea. Within the sensory patches, p75 expressing cells surrounded the outer hair cells (J). (K) Phalloidin staining of the whole cochlea from P4 embryos, showing complete differentiation of hair cells in both *Fgf20^βGal/+^* and *Fgf20^βGal/βGal^* mice.
(TIF)

Figure S4 Normal formation of the cochlear sensory domain in *Fgf20^βGal/βGal^* embryos. (A, B) Staining of the whole cochlea for Sox2 expression showing comparable expression patterns in *Fgf20^βGal/+^* (A) and *Fgf20^βGal/βGal^* (B) embryos at E13.5. (C, D) Staining of cochlear sections for Sox2 expression showing comparable expression patterns in *Fgf20^βGal/+^* (C) and *Fgf20^βGal/βGal^*(D) embryos at E14.5. (E, F) Staining of cochlear sections for Jag1 expression showing comparable expression patterns in *Fgf20^βGal/+^* (E) and *Fgf20^βGal/βGal^* (F) embryos at E14.5. (G, H) BrdU labeling of E14.5 cochlea showing comparable proliferation in *Fgf20^βGal/βGal^* (H) and *Fgf20^βGal/+^* (G) embryos.
(TIF)

Figure S5 Rescue of lateral compartment differentiation by FGF9. (A–K) Staining for Myo7a expression in *Fgf20^βGal/+^* and *Fgf20^βGal/βGal^* cochlear explants treated with or without FGF9. Treatment of *Fgf20^βGal/+^* explants with FGF9, either at E13.5 (B) or E16.5 (F), did not have any effect on hair cell number compared to untreated explants (A, E). Treatment of *Fgf20^βGal/βGal^* explants with FGF9 at E13.5 resulted in increased numbers of hair cells and decreased gaps between hair cell clusters (D) compared to untreated explants (C). Treatment of *Fgf20^βGal/+^* or *Fgf20^βGal/βGal^* explants with FGF9 at E16.5 did not affect hair cell number or the formation of gaps lacking sensory cells (G, H). (I) Quantitation of the number of hair cells in explants. The number of outer hair cells and total hair cells were rescued by treatment with FGF9 at E13.5 but not at E16.5. (J, K) Staining for Myo7a expression and BrdU incorporation in cochlear explants showing that Myo7a-stained hair cells do not co-label with BrdU, indicating that cells induced to differentiate in the gaps between sensory patches (arrow) differentiate in response to FGF9 without undergoing cell division.
(TIF)

## Abbreviations

ABR: auditory brainstem response
*βGal*: β-galactosidase
DCs: Deiters' cells
FGF: fibroblast growth factor
FGFR: FGF receptor
HC: hair cell
HeCs: Hensen's cells
IHC: inner hair cell
IPC: inner pillar cell
IPhC: inner phalangeal cells
OC: organ of Corti
OHC: outer hair cell
OPC: outer pillar cell
PCs: pillar cells
SC: supporting cell
