# Supplementary figures and images for: Differentiation of the Lateral Compartment of the Cochlea Requires a Temporally Restricted FGF20 Signal
Source: PLoS Biol. 2012 Jan 3;10(1):e1001231. doi: 10.1371/journal.pbio.1001231 (PMC3250500; doi:10.1371/journal.pbio.1001231)

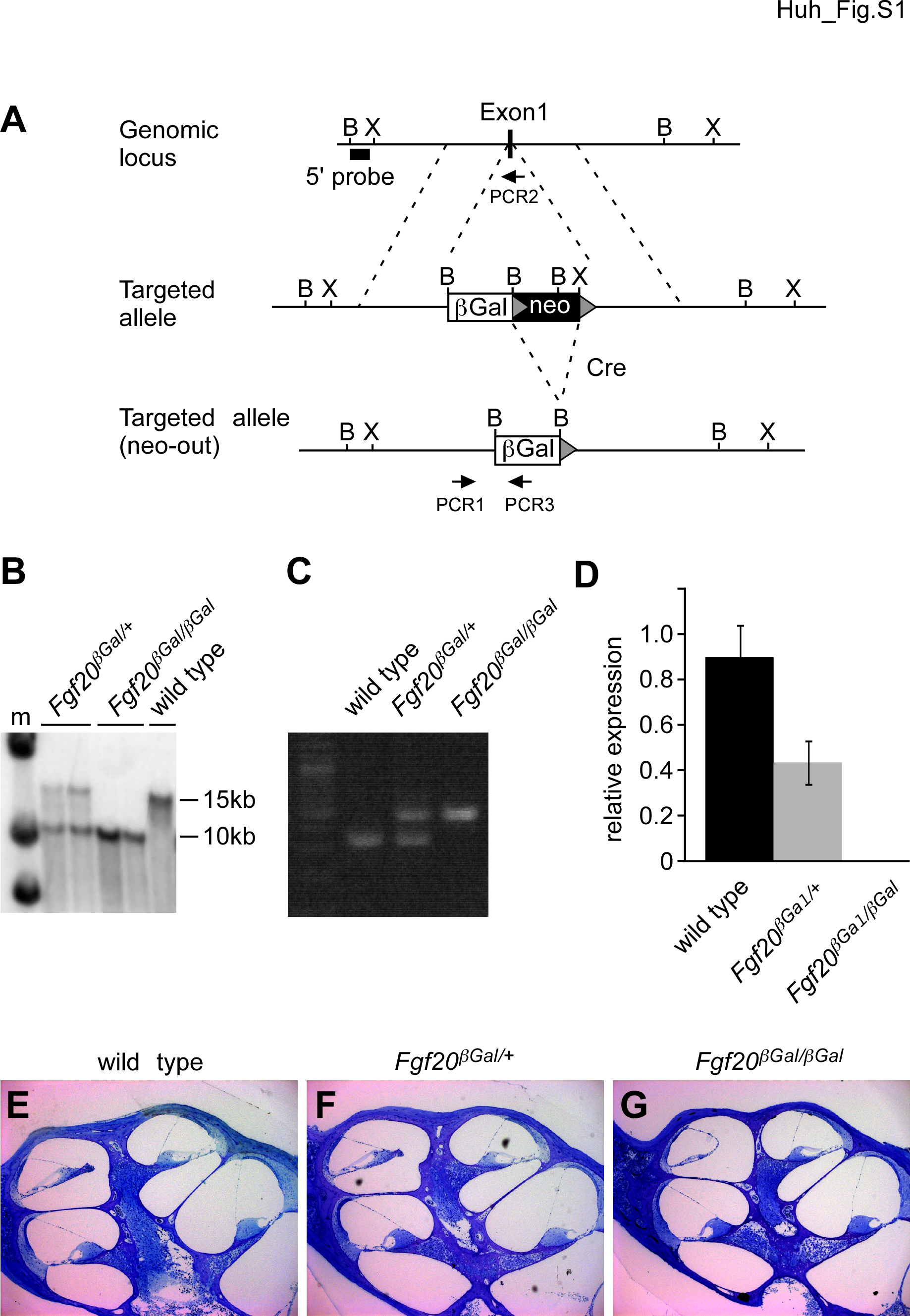

Supplement: Figure S1 — Fgf20 gene targeting and morphology of the adult cochlea. (A) Exon1 of the Fgf20 gene was replaced with a β-galactosidase gene and a PGK promoter-neomycin gene flanked by LoxP recombination sites. In vivo Cre mediated recombination (β-actincre) was used to excise the neomycin cassette. (B) Southern blot of wild type, Fgf20βGal/+, and Fgf20βGal/βGal mouse DNA digested with BamH1 and probed with a 5′ probe that is extrinsic to the targeting vector. Wild type 15 Kb and mutant 10 Kb bands are indicated. (C) PCR genotyping of the Fgf20βGal alleles showing wild type (335 bp) and mutant (498 bp) PCR fragments. Orientation of PCR primers is indicated. (D) Quantitative RT-PCR of E14.5 inner ear tissue showing expression of Fgf20 mRNA in wild type tissue, reduced expression in Fgf20βGal/+ tissue, and no detectable expression in Fgf20βGal/βGal tissue. (E–G) Thin sections stained with toluidine blue showing comparable cochlear morphology of 2-mo-old wild type (E), Fgf20βGal/+ (F), and Fgf20βGal/βGal(G) mice. (TIF) [file pbio.1001231.s001.tif]

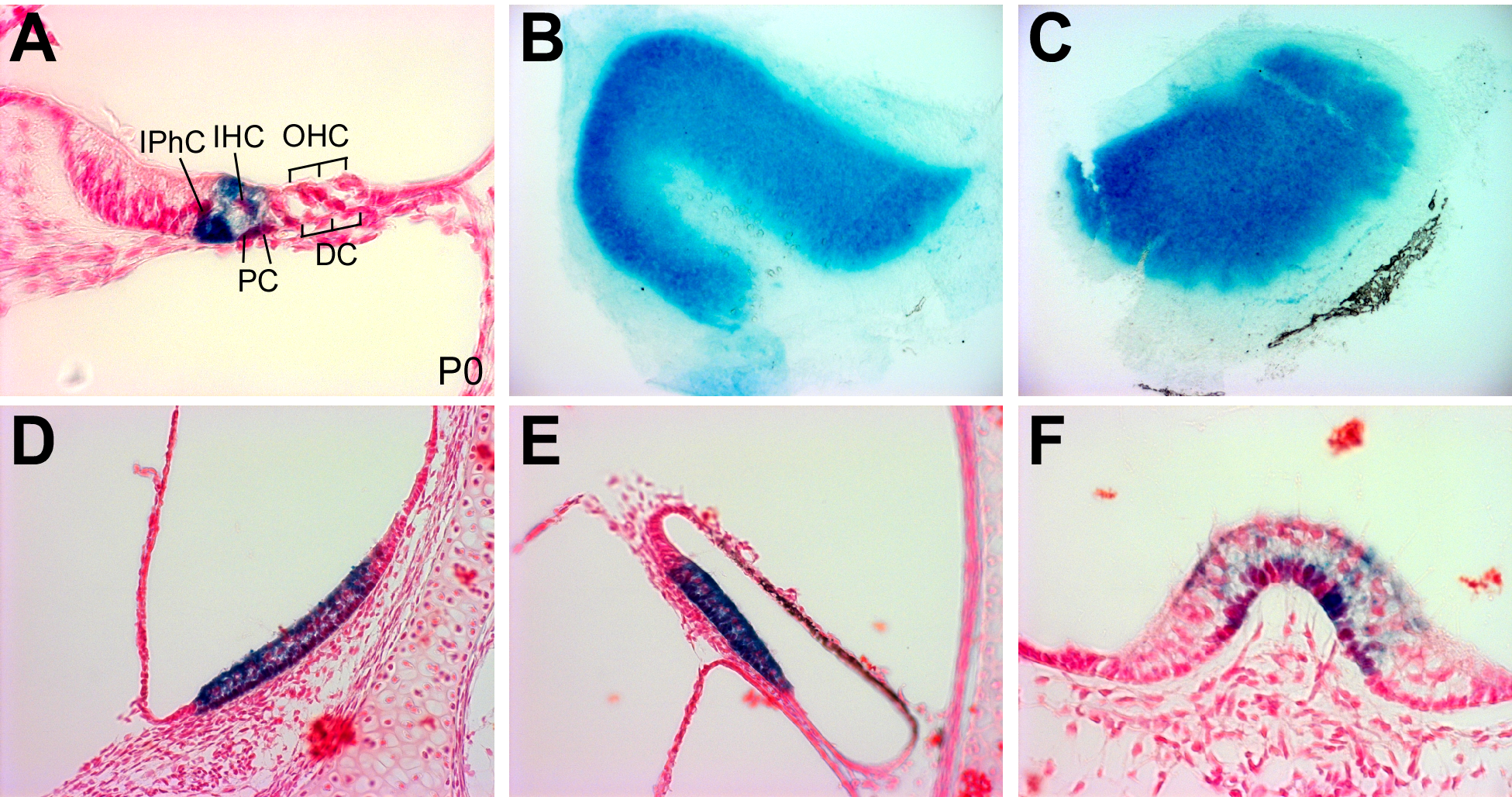

Supplement: Figure S2 — Expression of Fgf20 in the developing inner ear. (A–E) Whole mount (B, C) and sections (A,D–F) showing βGal expression in the sensory epithelium of the organ of Corti (A), utricle (B, D), saccule (C, E), and cristae of the semicircular canals (F) at P0. (TIF) [file pbio.1001231.s002.tif]

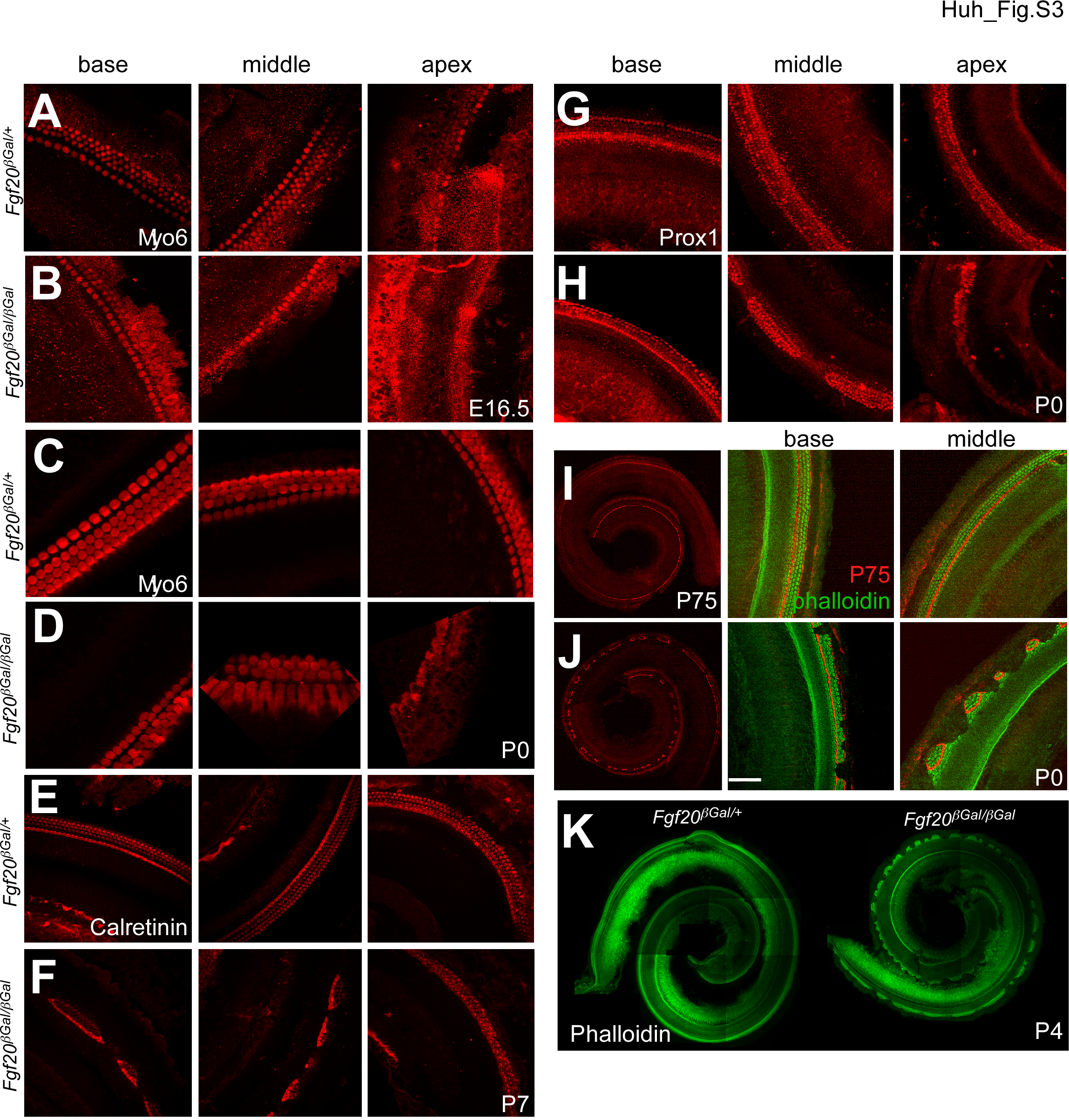

Supplement: Figure S3 — Hair cell and supporting cell formation in the mouse cochlea. (A,B) Staining of the cochlea with Myo6 expression, showing fewer differentiated hair cells towards the cochlear apex in Fgf20βGal/+ E16.5 embryos (A). In Fgf20βGal/βGal embryos, no distinctive phalloidin stained or Myo6 expressing hair cells were formed in the apical cochlea at E16.5 (B). (C, D) Staining of the P0 cochlea for Myo6 expression showing staining throughout the length of the cochlea in Fgf20βGal/+ embryos (C). Fgf20βGal/βGal embryos showed decreased Myo6 staining in the cochlear apex (D). (E, F) Staining of the P7 cochlea for Calretinin expression showing comparable expression levels in Fgf20βGal/+ (E) and Fgf20βGal/βGal (F) cochleae. (G, H) Staining of the cochlea for Prox1 expression showing two rows of pillar cells and three rows of Deiters' cells throughout the cochlea of Fgf20βGal/+ embryos (G). Fgf20βGal/βGal embryos had two rows of pillar cells and two rows of Deiters' cells in the base, patches of differentiated supporting cells containing two rows of pillar cells and three rows of Deiters' cells in the middle, and differentiated supporting cells in the apex (H). (I, J) Staining of the cochlea for p75 expression showing differentiated pillar cells (strong staining) and Henson's cells (weak staining) throughout the length of the cochlea of Fgf20βGal/+ embryos (I). Pillar cells and Henson's cells were also identified in Fgf20βGal/βGal cochlea, but the pattern matched that of hair cells, showing patches of differentiated cells and gaps of unlabeled cells in the middle region of the cochlea. Within the sensory patches, p75 expressing cells surrounded the outer hair cells (J). (K) Phalloidin staining of the whole cochlea from P4 embryos, showing complete differentiation of hair cells in both Fgf20βGal/+ and Fgf20βGal/βGal mice. (TIF) [file pbio.1001231.s003.tif]

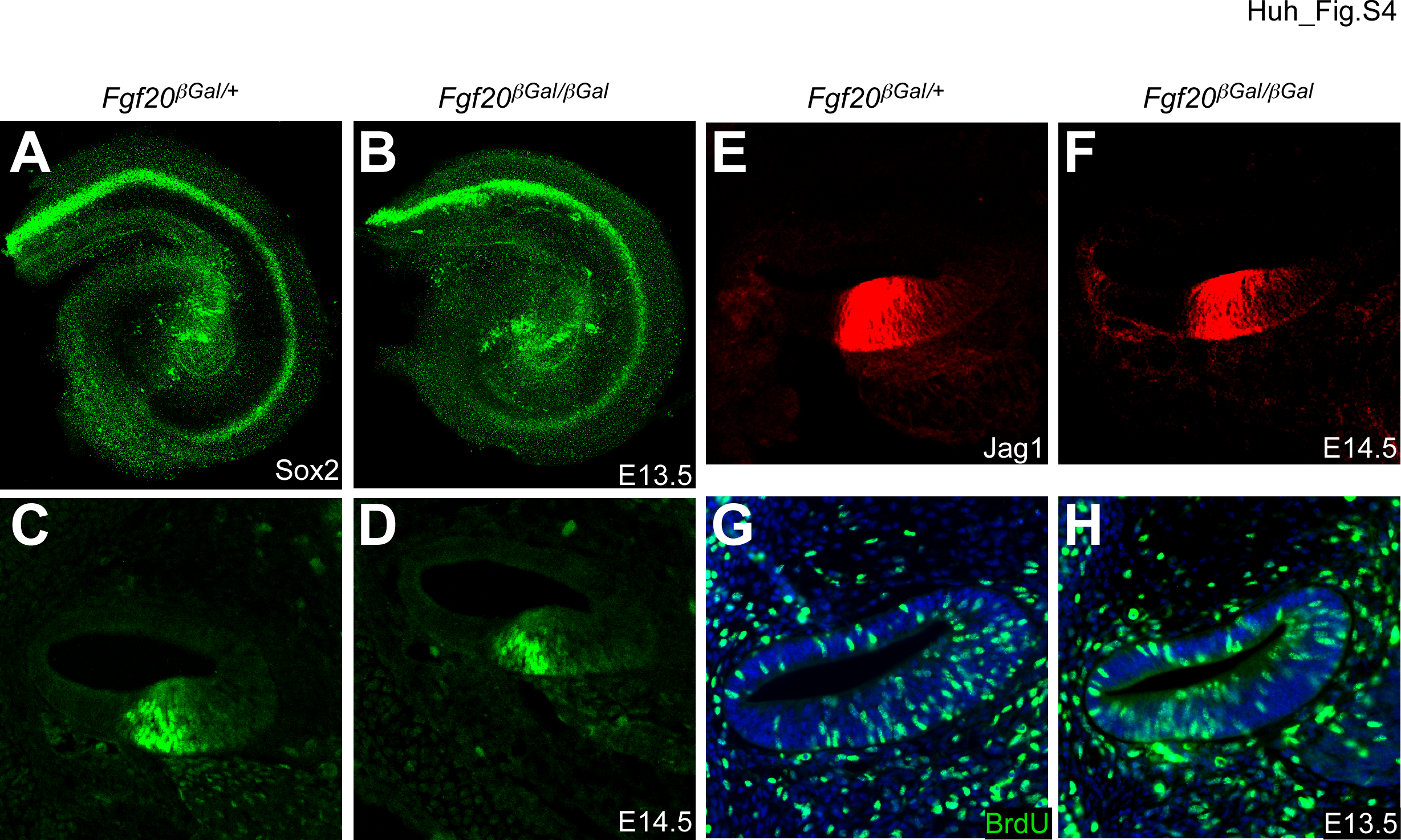

Supplement: Figure S4 — Normal formation of the cochlear sensory domain in Fgf20βGal/βGal embryos. (A, B) Staining of the whole cochlea for Sox2 expression showing comparable expression patterns in Fgf20βGal/+ (A) and Fgf20βGal/βGal (B) embryos at E13.5. (C, D) Staining of cochlear sections for Sox2 expression showing comparable expression patterns in Fgf20βGal/+ (C) and Fgf20βGal/βGal(D) embryos at E14.5. (E, F) Staining of cochlear sections for Jag1 expression showing comparable expression patterns in Fgf20βGal/+ (E) and Fgf20βGal/βGal (F) embryos at E14.5. (G, H) BrdU labeling of E14.5 cochlea showing comparable proliferation in Fgf20βGal/βGal (H) and Fgf20βGal/+ (G) embryos. (TIF) [file pbio.1001231.s004.tif]

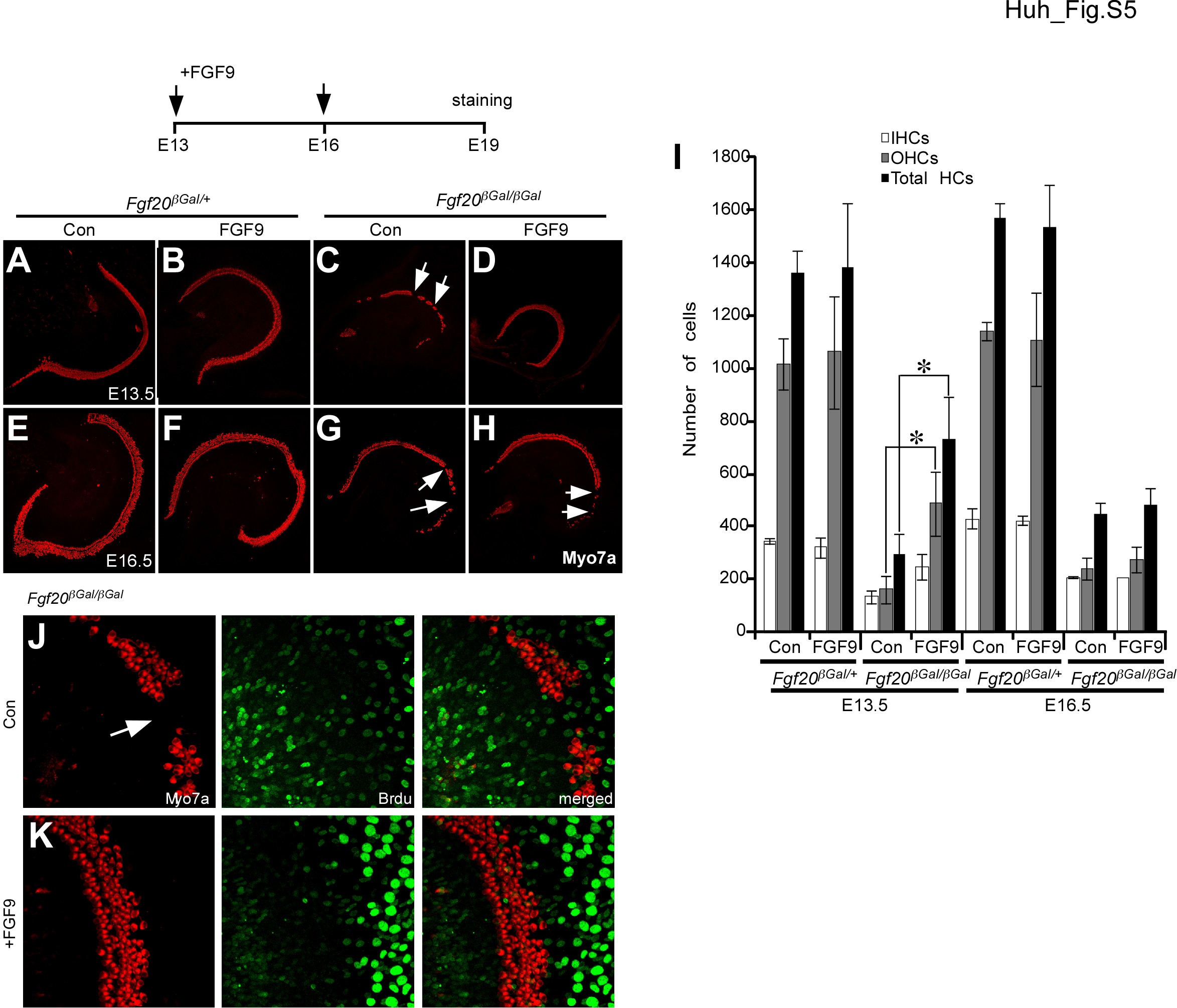

Supplement: Figure S5 — Rescue of lateral compartment differentiation by FGF9. (A–K) Staining for Myo7a expression in Fgf20βGal/+ and Fgf20βGal/βGal cochlear explants treated with or without FGF9. Treatment of Fgf20βGal/+ explants with FGF9, either at E13.5 (B) or E16.5 (F), did not have any effect on hair cell number compared to untreated explants (A, E). Treatment of Fgf20βGal/βGal explants with FGF9 at E13.5 resulted in increased numbers of hair cells and decreased gaps between hair cell clusters (D) compared to untreated explants (C). Treatment of Fgf20βGal/+ or Fgf20βGal/βGal explants with FGF9 at E16.5 did not affect hair cell number or the formation of gaps lacking sensory cells (G, H). (I) Quantitation of the number of hair cells in explants. The number of outer hair cells and total hair cells were rescued by treatment with FGF9 at E13.5 but not at E16.5. (J, K) Staining for Myo7a expression and BrdU incorporation in cochlear explants showing that Myo7a-stained hair cells do not co-label with BrdU, indicating that cells induced to differentiate in the gaps between sensory patches (arrow) differentiate in response to FGF9 without undergoing cell division. (TIF) [file pbio.1001231.s005.tif]
